# Cross-Sectional Analysis of Fruit and Vegetable Consumption, Breakfast Frequency, and Life Satisfaction Among Health Sciences Students: The Mediating Role of Positive Affect

**DOI:** 10.3390/nu18010122

**Published:** 2025-12-30

**Authors:** Jacksaint Saintila, Norma Del Carmen Gálvez-Díaz, Luz A. Barreto-Espinoza, Christian Casas-Gálvez, Ana Valle-Chafloque, Ramos Alfonso Paredes-Aguirre, Yaquelin E. Calizaya-Milla

**Affiliations:** 1 Research Group for Nutrition and Healthy Behaviors, School of Medicine, Universidad Señor de Sipán, Carretera a Pimentel Km 5, Lambayeque 14001, Peru; ncarmengd@gmail.com (N.D.C.G.-D.); labe@uss.edu.pe (L.A.B.-E.); cgalvezchristic@uss.edu.pe (C.C.-G.); valleana2911@gmail.com (A.V.-C.); 2 Escuela de Posgrado, Unidad de Posgrado de Salud, Universidad Peruana Unión, Lima 15, Peru; raparedesag1957@ucvvirtual.edu.pe; 3 School of Human Nutrition, Universidad Peruana Unión, Lima 15, Peru; yaquelincalizaya@upeu.edu.pe

**Keywords:** breakfast, feeding behavior, psychological well-being, personal satisfaction, vegetables, fruit, universities, Peru

## Abstract

**Background**: Eating habits such as fruit and vegetable (FV) consumption and breakfast frequency are well recognized for their contribution to overall health and well-being. However, the psychological mechanisms that explain the link between these habits and life satisfaction remain poorly explored among university students in the health sciences. **Objective:** To examine whether positive affect mediates the relationship between FV consumption, breakfast frequency, and life satisfaction among health sciences students. **Methods**: A cross-sectional study was conducted with 511 students. FV consumption, breakfast frequency, positive affect, and life satisfaction were assessed using self-report measures. Mediation models were applied to estimate direct and indirect associations. **Results:** FV consumption and breakfast frequency were positively associated with both positive affect and life satisfaction. Although the direct associations with life satisfaction were not significant, the indirect associations through positive affect were significant (FV: β = 0.114, 95% CI [0.055, 0.173]; breakfast: β = 0.133, 95% CI [0.073, 0.192]). The model accounted for 51.4% of the variance in life satisfaction. **Conclusions:** The results highlight the role of positive affect as a psychological mechanism linking everyday eating habits to life satisfaction, emphasizing the need to integrate emotional components into strategies for promoting healthy lifestyles among university populations.

## 1. Introduction

Regular consumption of fruits and vegetables (FV), along with the habitual practice of eating breakfast, is among the eating behaviors considered essential markers of a healthy diet and represents a fundamental pillar for emotional well-being [1,2,3]. Despite this evidence, adherence to these recommendations has been reported as insufficient among university students. In several low- and middle-income countries, approximately 80% of university and medical students fail to meet the World Health Organization (WHO) recommendations for fruit and vegetable intake [4,5], while in Peru, more than half of university students report insufficient FV consumption [6].

Regarding breakfast, previous studies conducted in diverse university settings have consistently reported high rates of irregular intake among health sciences students, indicating that this habit remains insufficiently adopted worldwide [7,8]. In Peru, previous findings indicate that although medical students tend to have a higher frequency of regular breakfast consumption than non-medical students, overall adherence remains far from optimal [9]. This reality is particularly concerning among health sciences students, who not only need to maintain healthy lifestyles for their own well-being but will also be future professionals responsible for promoting health among the population.

Life satisfaction is one of the most important and widely studied indicators of psychological well-being. It is defined as the cognitive evaluation a person makes regarding the overall quality of their life, reflecting a sense of fulfillment and contentment [10]. Among health sciences students, greater life satisfaction is associated with better mental health outcomes, including lower levels of depression, anxiety, and stress, as well as higher psychological well-being [11]. However, life satisfaction is part of a broader construct known as subjective well-being, which comprises two fundamental dimensions: a cognitive one, represented by life satisfaction, and an affective one, related to the experience of positive emotions [12]. In the present study, although subjective well-being is considered the overarching conceptual framework, its two dimensions were examined separately. Life satisfaction was used as the main outcome variable, given its theoretical relevance and its utility in evaluating global perceptions of well-being [13,14], while positive affect was analyzed as a mediating mechanism, based on previous evidence linking it to the influence of eating habits on well-being [15,16].

Evidence specifically linking FV consumption with life satisfaction among university students in the health sciences is limited. Most available studies focus on broader indicators of well-being—such as happiness, positive affect, and general mental health—rather than life satisfaction itself. For example, studies among medical students in Iran and other university populations have shown that higher FV intake is associated with more days of happiness, greater energy, and fewer days of poor mental health [17,18], while another study found that fruit and vegetable intake was positively associated with positive affect, but showed no significant association with life satisfaction in a general university sample [19]. These findings suggest a positive association between FV intake and well-being, although direct evidence regarding life satisfaction among health sciences students remains scarce.

A similar pattern is observed for breakfast consumption. Research has consistently linked regular breakfast intake with positive emotional functioning and general psychological well-being among university students, including those in the health sciences. Studies indicate that eating breakfast daily is associated with greater happiness [17], while skipping breakfast is linked to higher levels of depressive symptoms [20,21]. Despite this, most studies emphasize general well-being or quality of life rather than life satisfaction specifically, leaving direct evidence in health sciences students limited.

Positive affect has been proposed as a fundamental psychological mechanism in the relationship between healthy eating habits and subjective well-being. From a theoretical perspective, it has been suggested that positive emotions broaden cognitive and behavioral repertoires, foster resilience, and promote more favorable evaluations of life quality, as described in Fredrickson’s broaden-and-build theory [22]. Likewise, self-determination models propose that positive affect arises from behaviors that satisfy basic needs for autonomy, competence, and self-care [23], which aligns with the adoption of healthy eating practices. In fact, research grounded in self-determination theory (SDT) consistently demonstrates that when individuals feel autonomous (acting by choice), competent (effective in their actions), and supported (connected to others), they experience more positive emotions and are more likely to adopt and maintain healthy behaviors, including a balanced diet [24,25]. In the present study, we therefore conceptualize positive affect as an affective mediator and life satisfaction as a cognitive outcome within the broader framework of subjective well-being, proposing that regular FV consumption and the habitual practice of eating breakfast may foster positive emotions that, in turn, are associated with higher life satisfaction.

Several studies on this topic partially support this hypothesis. Longitudinal research has shown that higher fruit and vegetable intake is associated with subsequent increases in positive affect and life satisfaction, suggesting that emotional processes may contribute to the link between diet and subjective well-being [26]. Furthermore, studies conducted among Spanish adults have reported that life satisfaction fully mediated the relationship between fruit and vegetable intake and happiness, which indicates that cognitive evaluations of one’s life may also play a role in how dietary behaviors relate to emotional outcomes [27]. Similarly, other studies have suggested that healthy dietary patterns—such as adherence to the Mediterranean diet or regular fruit consumption—are associated with higher levels of positive affect and life satisfaction [28,29], reinforcing the idea that both affective and cognitive components of subjective well-being tend to be more favorable in individuals with healthier eating habits.

However, although both positive affect and life satisfaction are related to healthy eating behaviors, they may function as independent predictors rather than as part of a strictly mediated process [28]. This suggests that, while positive affect can be considered an intermediate mechanism, there is still insufficient clarity regarding its specific role within the relationship between eating habits and psychological well-being.

This study arises from the need to achieve a more integrated understanding of the links between eating habits and psychological well-being among health sciences students. This group represents a meaningful population not only because they are frequently exposed to high academic stress and irregular schedules, but also because they are future health professionals who are expected to model and promote healthy lifestyle behaviors in their clinical and community practice. Although previous research has associated certain dietary behaviors with positive affective states or greater life satisfaction, few studies have simultaneously examined FV consumption and breakfast frequency within a single model, and even fewer have explicitly incorporated positive affect as an affective mediator and life satisfaction as a cognitive outcome. Addressing this gap is relevant not only for its conceptual value—by clarifying the psychological mechanisms that may underlie eating behaviors—but also for its practical implications in designing strategies to promote healthy lifestyles in the university setting. Within this framework, the present study aimed to analyze the relationship between fruit and vegetable consumption, breakfast frequency, and life satisfaction, considering the mediating role of positive affect within the broader construct of subjective well-being among health sciences students.

## 2. Materials and Methods

### 2.1. Design and Participants

A cross-sectional study was conducted with health sciences students enrolled in technical programs from two higher education institutions in the Lambayeque region, Peru. Participants were selected through non-probabilistic convenience sampling, which may limit the generalizability of the findings beyond the study population. Inclusion criteria comprised students enrolled in a health sciences technical program, of both sexes, aged between 18 and 30 years, who voluntarily agreed to participate and provided informed consent. Questionnaires that were incomplete or contained inconsistent responses (defined as contradictory answers to related items, uniform response patterns across all items, or missing data exceeding 10%) were excluded, as well as those from participants who were pregnant, ill, or under 18 years of age.

The sample size was estimated using the *semPower* package (version 2.0) in R (version 4.3.1) [30], based on an RMSEA-driven power analysis for structural equation modeling. A target RMSEA value of 0.05, corresponding to a close model fit, was specified, together with a statistical power of 0.95 and a significance level of α = 0.05. The power estimation was conducted assuming a mediation model including two observed exogenous variables (fruit and vegetable consumption and breakfast frequency), one latent mediator (positive affect), and one latent outcome (life satisfaction). Under these specifications, the minimum recommended sample size was approximately 320 participants. To ensure adequate power and stable parameter estimation, particularly for indirect associations, the final analytical sample included 511 students, exceeding commonly recommended thresholds for SEM analyses.

### 2.2. Procedure

Data collection was conducted in person in classroom settings and designated campus areas (e.g., common academic spaces) within the participating institutions, during scheduled academic activities. The questionnaire included an initial section describing the study and the informed consent, followed by sociodemographic questions and the corresponding measurement instruments. Participation was anonymous, voluntary, and without economic or academic incentives. Trained interviewers, who were not informed of the specific study hypotheses, explained the general objectives of the study, answered questions, and ensured that each participant provided explicit consent before beginning the survey. The average completion time was approximately 10 min. The research protocol was approved by the Ethics Committee of the Universidad Señor de Sipán (Ref.: 1328-CIEI, on 29 August 2025) and was carried out in accordance with the ethical principles established in the Declaration of Helsinki.

### 2.3. Instruments and Variables

The short version of the Subjective Well-Being Scale (EBS-8) developed by Calleja and Mason [12] was used. This scale consists of eight items derived from the original 20-item version (EBS-20) and is organized into two dimensions: life satisfaction (4 items; e.g., “I like my life”) and positive affect (4 items; e.g., “I am a happy person”). Each item is rated on a 6-point Likert scale ranging from 1 = “Strongly disagree” to 6 = “Strongly agree.” The EBS-8 has demonstrated adequate psychometric properties in university populations, with high internal consistency reported for life satisfaction (α = 0.948) and positive affect (α = 0.964) in the original validation study [12]. In the present study, both dimensions were analyzed separately within the framework of subjective well-being and showed satisfactory internal consistency (positive affect: α = 0.852; life satisfaction: α = 0.912).

FV consumption was assessed using a structured self-report question on daily servings. The WHO recommendation of ≥5 servings/day was used as a reference indicator of healthy intake [31]. However, because only a small proportion of participants met this threshold, FV intake was categorized into multiple levels (none, 1, 2, 3–4, ≥5 servings/day) exclusively for descriptive purposes (Table 1). Accordingly, higher FV intake categories represent relative consumption levels within the sample rather than strict adherence to WHO guidelines. For correlational analyses and structural equation modeling, the original FV variable was retained and modeled as a continuous observed variable to preserve variability and statistical power. This self-report method has been used and validated in previous studies with university populations and is considered an acceptable indicator of compliance with dietary guidelines [32].

Breakfast habits were assessed through a single question: “How many days per week do you usually eat breakfast?” Participants indicated a value between 0 and 7, corresponding to the number of days they typically consumed this meal. Based on their responses, three frequency categories were established: low (0–2 days/week), moderate (3–5 days/week), and high (6–7 days/week). These thresholds were chosen to distinguish between irregular breakfast consumption, occasional intake during the week, and regular daily or near-daily breakfast habits, a categorization commonly applied in studies involving university populations. This classification method has been used in previous research and has demonstrated adequate applicability [33,34]. For the purposes of the structural equation modeling, this categorical variable was subsequently recoded into an ordered numeric scale (1 = low, 2 = moderate, 3 = high) to represent increasing levels of breakfast frequency and was included in the model as an observed ordinal variable.

### 2.4. Statistical Analysis

Data were processed and analyzed using the Statistical Package for the Social Sciences (SPSS) version 25 for descriptive statistics and bivariate analyses, while mediation analyses were conducted using structural equation modeling (SEM) implemented in JASP software (version 0.18.3). Descriptive analyses included means, standard deviations, coefficients of variation, and skewness and kurtosis indices to summarize the distributions of the study variables. Skewness and kurtosis values within ±2 were considered acceptable for descriptive purposes.

Pearson correlation analyses were conducted to examine bivariate associations among fruit and vegetable consumption, breakfast frequency, positive affect, and life satisfaction. Internal consistency of the positive affect and life satisfaction scales was assessed using Cronbach’s alpha coefficient. Convergent validity was evaluated using the Average Variance Extracted (AVE), with values greater than 0.50 considered indicative of adequate convergent validity.

Prior to testing the structural model, confirmatory factor analyses (CFA) were conducted to evaluate the measurement models of positive affect and life satisfaction. Model adequacy was assessed using multiple goodness-of-fit indices, including the comparative fit index (CFI), Tucker–Lewis index (TLI), root mean square error of approximation (RMSEA), and standardized root mean square residual (SRMR), following commonly recommended cut-off criteria.

As an initial exploratory diagnostic, variance inflation factors (VIF) and tolerance indices were examined at the observed-variable level to assess potential collinearity among the predictors. In addition, collinearity in the SEM context was evaluated through the inspection of latent variable correlations and evidence of discriminant validity, as reflected by AVE values exceeding recommended thresholds. Together, these assessments suggested that multicollinearity was unlikely to substantially bias the estimated associations.

In the structural models, fruit and vegetable consumption and breakfast frequency—originally assessed as ordinal categories—were treated as ordered indicators. Positive affect was specified as the mediating variable in the association between eating habits and life satisfaction. Direct, indirect, and total associations were estimated using 95% confidence intervals based on bootstrap resampling with 5000 iterations. All path coefficients are reported as completely standardized estimates. A significance level of *p* < 0.05 was adopted for all analyses.

## 3. Results

A total of 511 health sciences students participated in the study. The sample consisted of 64.8% women (*n* = 331) and 35.2% men (*n* = 180), with a mean age of 20.24 years (SD = 2.61). Most participants were single (79.8%) and came from the coastal region of the country (76.1%). Nearly half of the students reported working while studying (48.0%). Most participants reported consuming only one serving (33.66%) or two servings (32.09%) of fruit per day. Only about one-fifth of the sample (21.33%) reported a high FV intake, defined as three or more servings daily. Regarding breakfast frequency, 59.49% of participants reported eating breakfast regularly (6–7 days per week). However, 40.51% of the sample showed an irregular or absent breakfast habit, eating breakfast five or fewer days per week.

Table 2 presents the results for average fruit consumption, which ranged between two and three servings per day (M = 2.66, SD = 1.03). The mean frequency of breakfast consumption was high, approaching the regular category (M = 2.45, SD = 0.73). Likewise, levels of positive affect were moderately high (M = 15.88, SD = 4.31), while life satisfaction showed a mean value close to the midpoint of the scale (M = 13.95, SD = 5.15), representing the greatest variability among all variables. The assessment of univariate normality assumptions through skewness and kurtosis indices indicated that all values were within acceptable ranges (±2), suggesting that the distributions did not deviate substantially from normality. It should be noted that the mean values reported for fruit and vegetable consumption and breakfast frequency correspond to ordinally coded variables used for analytical purposes (FV: 1 = none to 5 = ≥5 servings/day; breakfast: 1 = 0–2 days/week to 3 = 6–7 days/week). These means do not represent exact daily intake or frequency but reflect relative positions within the ordered response categories.

Table 3 presents the correlations, which showed that all eating habits (fruit consumption and breakfast frequency) were positively and significantly correlated with both measures of well-being (positive affect and life satisfaction), with correlation coefficients ranging from r = 0.17 to r = 0.22, indicating weak associations according to conventional effect size criteria. Furthermore, a strong and positive correlation was observed between positive affect and life satisfaction (r = 0.715, *p* < 0.001). The positive affect (α = 0.852) and life satisfaction (α = 0.912) scales demonstrated excellent internal consistency. In addition, convergent validity analyses, measured through the Average Variance Extracted (AVE), confirmed that both scales had adequate validity (AVE > 0.50). Finally, verification of the no-multicollinearity assumption through the calculation of Variance Inflation Factors (VIF) yielded values very close to 1 (between 1.066 and 1.077) and tolerance indices also near 1 (between 0.929 and 0.938), which are well below the critical thresholds of 10 and 0.10, respectively. This confirms that no multicollinearity was present that could bias the results of the mediation model.

### Model Fit Indices

The structural equation model showed an excellent fit to the data. The comparative fit index (CFI = 1.00) and the Tucker–Lewis index (TLI = 1.00) exceeded the recommended cutoff values (>0.95). The root mean square error of approximation was negligible (RMSEA = 0.00, 90% CI [0.00, 0.03], *p* = 0.968), and the standardized root mean square residual was very low (SRMR = 0.001). Together, these indices indicate that the proposed mediation model adequately represents the observed data.

**Figure 1:**
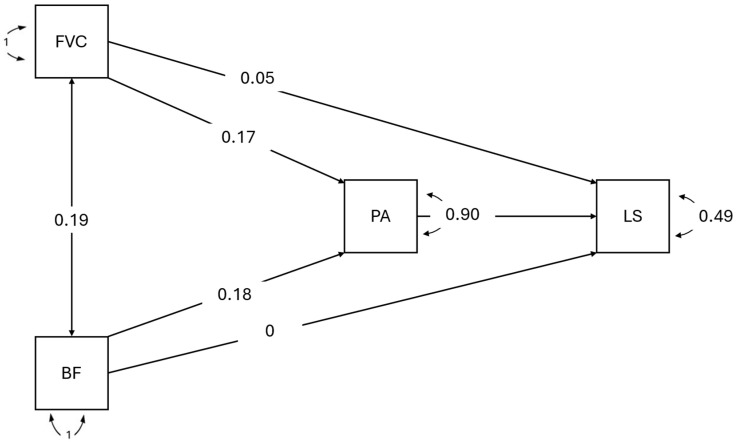
Structural Model of Eating Habits, Positive Affect, and Life Satisfaction. ***Note.*** FVC: Fruit and Vegetable Consumption; BF: Breakfast Frequency; PA: Positive Affect; LS: Life Satisfaction. Path coefficients represent completely standardized estimates.

All coefficients reported in the mediation analysis correspond to completely standardized estimates, allowing direct comparison of effect sizes across paths. Table 4 shows that the direct associations between both eating habits and life satisfaction, after controlling for positive affect, were not statistically significant (fruit consumption: β = 0.047, *p* = 0.128; breakfast frequency: β = 0.005, *p* = 0.876) (see Figure 1). In contrast, the indirect associations estimated through positive affect were statistically significant, indicating that fruit consumption was indirectly associated with life satisfaction via positive affect (β = 0.114, 95% CI [0.055, 0.173]). Similarly, breakfast frequency also showed a significant indirect association (β = 0.133, 95% CI [0.073, 0.192]). Regarding the associations between fruit and vegetable consumption, breakfast frequency, and positive affect, the standardized coefficients were statistically significant but small in magnitude (β ≈ 0.16–0.19), indicating modest associations.

The explanatory capacity of the model, as indicated by the coefficients of determination (R^2^), showed that fruit consumption, breakfast frequency, and positive affect were jointly associated with a substantial proportion of the variance in life satisfaction (R^2^ = 0.514). This indicates that more than 50% of the variability in life satisfaction was statistically accounted for by the variables included in the model. In addition, fruit consumption and breakfast frequency were associated with a smaller, yet statistically significant, proportion of the variance in positive affect (R^2^ = 0.071), reflecting a small effect size.

## 4. Discussion

The results of the present study indicate that the relationship between eating habits—specifically FV consumption and breakfast frequency—and life satisfaction is fully mediated by positive affect. Although neither of the evaluated habits showed a significant direct association with life satisfaction, both exhibited robust indirect associations through positive affect, suggesting that positive emotions constitute the psychological mechanism that may explain how these eating behaviors are linked to overall well-being. Although the indirect associations through positive affect were statistically significant, their magnitude was modest. Therefore, these findings should be interpreted as indicative of potential associative pathways rather than definitive mediational processes.

First, the results showed that neither fruit and vegetable consumption nor breakfast frequency was directly associated with life satisfaction once positive affect was included in the model. This suggests that engaging in these eating habits alone does not immediately translate into a more positive global evaluation of one’s life. This finding is consistent with the literature indicating that dietary behaviors, although relevant for physical health, tend to influence subjective well-being through more immediate emotional and psychological processes rather than exerting a direct association [28,29]. Likewise, since life satisfaction represents a more stable cognitive judgment and is less reactive to isolated daily behaviors [35,36]—although it may show some short-term variability in certain individuals, particularly following recent changes in emotional state or immediate life experiences [35,37]—it is expected that specific habits such as fruit and vegetable consumption or breakfast regularity may not, by themselves, produce sufficient changes to modify this overall evaluation.

In contrast to the absence of direct associations, both eating habits showed significant indirect associations with life satisfaction through positive affect. This pattern is consistent with Fredrickson’s broaden-and-build theory, which posits that positive emotions expand individuals’ cognitive and behavioral resources, facilitating processes such as positive life evaluation [22]. It also aligns with Diener’s classical models of subjective well-being [38,39], which position positive affect as a fundamental component of well-being—both for its direct contribution and its facilitating role in other domains of human functioning [40]. Therefore, these findings suggest that the eating habits examined modestly contribute to daily emotional well-being, and that these positive affective states, in turn, promote greater life satisfaction. Thus, positive affect functions as a psychological bridge linking everyday eating behaviors to broader evaluations of personal well-being.

Another relevant finding of the present study is that fruit and vegetable consumption showed a positive and significant, though modest, relationship with positive affect. This result is consistent with previous research linking higher intake of fresh, micronutrient-rich foods with better affective states [19,41]. Specifically, among health sciences university students, fruit and vegetable consumption has been associated with higher levels of happiness [17]. Prior experimental and observational literature suggests that plant-based foods, rich in vitamins, antioxidants, and bioactive compounds, may support emotional regulation through neurophysiological and anti-inflammatory pathways [42,43]. For instance, antioxidants and polyphenols have been associated with reduced oxidative stress and neuroinflammation and with neuroprotective effects in experimental contexts, potentially influencing serotonergic and dopaminergic activity [43,44]. Although these mechanisms were not directly assessed in the present study, the observed association aligns with existing literature suggesting a potential role of fruit- and vegetable-rich diets in supporting positive affective experiences.

Similarly, breakfast frequency was positively associated with positive affect. Although research specifically addressing positive affect is limited, prior studies have reported positive associations between regular breakfast consumption and indicators of happiness, attention, and emotional well-being in university populations [17,20,45,46].

From a theoretical perspective, regular breakfast consumption has been associated with greater glycemic stability and improved cognitive functioning, which may support emotional regulation and daily affective balance [47,48]. Some studies have suggested that omitting breakfast may be associated with heightened physiological stress responses and altered hormonal regulation, including cortisol secretion, particularly during the morning hours [49,50,51]. While these biological and behavioral pathways cannot be confirmed within the present cross-sectional design, they provide a plausible theoretical framework for interpreting the observed associations.

On the other hand, positive affect showed a strong and significant association with life satisfaction; it is likely that students who experience pleasant emotions more frequently tend to evaluate their lives more favorably. These findings are consistent with previous literature that has identified positive affect as one of the key predictors of life satisfaction. For instance, studies among medical students have found that life satisfaction was significantly and positively associated with positive affect [52]. Similarly, in another group of medical students, positive emotions were significantly associated with life satisfaction, even after accounting for other factors such as academic self-efficacy and self-regulated learning [53]. Comparable results have been reported among nursing and other health science students, where higher positive affect [54] and positive emotional states—such as optimism and happiness—were linked to greater life satisfaction and better adaptation to academic and social challenges [55]. In our study, a high coefficient (β ≈ 0.705) was observed. Likewise, previous research indicates that positive affect often shows a stronger association with life satisfaction than negative affect or other well-being factors, and that this relationship remains robust across different age groups and cultural contexts [56,57]. It is important to note that positive affect contributes to life satisfaction by enhancing coping skills, resilience, and social relationships—factors that are particularly important in the demanding context of health science education [52,53]. Therefore, maintaining positive affective states constitutes a decisive factor for preserving life satisfaction and, consequently, the overall well-being of students.

### 4.1. Limitations and Future Considerations

Among the main limitations of the present study is the use of a cross-sectional design, which prevents the establishment of causal relationships between eating habits, positive affect, and life satisfaction. Additionally, fruit and vegetable consumption and breakfast frequency were assessed using single-item self-report questions. While this approach facilitates data collection in large samples, it may limit measurement accuracy and reduce the ability to capture variability in these behaviors, potentially introducing recall bias and social desirability effects. Another aspect to consider is that the use of simplified, single-item measures for dietary behaviors may have constrained the precision of the estimates, as more comprehensive dietary assessment tools could provide a richer characterization of eating patterns. Furthermore, the sample consisted of students from only two higher education institutions in the Lambayeque region, which restricts the generalization of the results to other educational or cultural contexts. Finally, potentially influential variables related to well-being—such as sleep quality, physical activity, or academic stress—were not included, although these factors may interact with eating habits in their association with positive affect.

Based on these limitations, future research should explore these relationships using longitudinal or experimental designs that allow for the examination of causal and directional associations among the variables. It would also be advisable to include more precise and multidimensional measures of eating habits, taking into account the quality, diversity, and timing of food consumption. From a psychological perspective, future studies should incorporate additional emotional variables such as negative affect, emotion regulation, or resilience, to broaden the understanding of the mechanisms linking diet to subjective well-being. Finally, it is recommended to replicate the proposed model in other university populations and among health professionals to assess the consistency of the findings and strengthen the external validity of the model.

### 4.2. Theoretical and Practical Contributions

Despite the aforementioned limitations, this study provides a relevant theoretical contribution by proposing a model that integrates everyday eating habits—such as fruit and vegetable consumption and breakfast frequency—with emotional well-being. The findings demonstrate that the positive associations of eating habits with life satisfaction may occur primarily through positive affect. This approach broadens the traditional understanding of nutrition by incorporating psychological dimensions into the explanation of the benefits of a balanced diet.

From a practical perspective, the results highlight the need for university mental health and well-being programs to incorporate nutrition-sensitive emotional pathways, rather than addressing eating behaviors and mental health as independent targets. Specifically, interventions that promote regular breakfast consumption and higher fruit and vegetable intake may serve as low-cost, scalable strategies to support daily positive affect, which in turn contributes to greater life satisfaction.

Such integrated programs could include components such as gratitude practices, physical activity, and mindful eating, embedded within existing student mental health services, and could be implemented through interdisciplinary workshops, personalized counseling, and institutional campaigns focused on emotional and nutritional self-care. This would be particularly beneficial and relevant for health science students, for whom preventive, lifestyle-based mental health strategies are especially pertinent, given their high academic demands, significant stress levels, and increased emotional vulnerability.

## 5. Conclusions

The results of this study show that positive affect plays a mediating role in the relationship between eating habits (FV consumption and breakfast frequency) and life satisfaction among health science students. The relationship between fruit and vegetable intake, breakfast frequency, and subjective well-being is not direct but is primarily explained through the experience of daily positive emotions. This finding reinforces the notion that healthy eating behaviors impact not only physical health but also emotional and psychological well-being. Therefore, the results suggest that universities should promote strategies for emotional and physical well-being that include nutrition education, the creation of environments that facilitate healthy food choices, and the implementation of programs aimed at strengthening positive affect. Furthermore, this study opens new perspectives for research on the relationship between diet and well-being, adopting a multidimensional approach that integrates biological, psychological, and social aspects.

## Figures and Tables

**Table 1:** Eating Habits: FV Consumption and Breakfast Frequency.

Variables/Categories	*n*/M ± SD	%
**Sex**		
Male	180	35.2
Female	331	64.8
**Age (years)**	20.24 ± 2.61	**-**
**Marital status**		
With partner	103	20.2
Without partner	408	79.8
**Region of origin**		
Coast	389	76.1
Highlands	65	12.7
Jungle	57	11.2
**Works while studying**		
Yes	264	51.7
No	66	12.9
**Fruit and vegetable consumption**		
None	66	12.9
1 serving/day	172	33.7
2 servings/day	164	32.1
3–4 servings/day	90	17.6
≥5 servings/day	19	3.7
**Breakfast frequency**		
Rarely or never (0–2 days per week)	72	14.1
Occasional (3–5 days per week)	135	26.4
Regular (6–7 days per week)	304	59.5

***Note*.** M ± SD: mean and standard deviation. Fruit and vegetable categories reflect relative intake levels within the sample. Although the WHO recommends ≥5 servings/day, very few participants met this criterion.

**Table 2:** Descriptive Statistics and Normality of Study Variables.

Variables	M	SD	CV	Sk	Ku	Min	Max
1. Fruits and Vegetables	2.656	1.03	0.388	0.22	−0.556	1	5
2. Breakfast	2.454	0.729	0.297	−0.939	−0.52	1	3
3. Positive affect	15.883	4.306	0.271	0.314	−0.732	4	24
4. Life satisfaction	13.953	5.15	0.369	0.635	−0.569	4	24

***Note.*** Mean (M), Std. Deviation (SD), Coefficient of variation (CV), Skewness (Sk), Kurtosis (Ku), Minimum (Min), Maximum (Max). Means for fruit and vegetable consumption and breakfast frequency reflect ordinally coded response categories rather than continuous intake measures.

**Table 3:** Correlation Analysis, Reliability, and Collinearity Diagnostics.

Variable	1	2	3	4	α	Collinearity Statistics		AVE
						Tolerance	VIF	
1. Fruits and Vegetables	1	0.198	0.199	0.189	-	0.938	1.066	-
2. Breakfast	0.198	1	0.22	0.17	-	0.933	1.072	-
3. Positive affect	0.199	0.22	1	0.715	0.852	0.929	1.077	0.629
4. Life satisfaction	0.189	0.17	0.715	1	0.912	-	-	0.738

***Note*.** α = Cronbach’s alpha; AVE = Average Variance Extracted; VIF = Variance Inflation Factor. Tolerance and VIF values were used to assess multicollinearity among predictors, while AVE values (>0.50) indicate adequate convergent validity of the latent constructs. Although the correlation between positive affect and life satisfaction was high (r = 0.715), this magnitude is consistent with previous research conceptualizing these variables as closely related yet distinct affective and cognitive components of subjective well-being. The AVE values exceeding the recommended threshold for both constructs support their discriminant validity, indicating that each latent variable captured sufficient unique variance.

**Table 4:** Results of the mediation analysis of the variables.

Effect	β	SE	z-Value	*p*	95% CI
**Direct effects**					
Fruits and Vegetables → Life satisfaction	0.047	0.031	1.524	0.128	[−0.014, 0.109]
Breakfast frequency → Life satisfaction	0.005	0.033	0.155	0.876	[−0.06, 0.071]
**Indirect effects**					
Fruits and Vegetables → Positive affect → Life satisfaction	0.114	0.03	3.796	<0.001	[0.055, 0.173]
Breakfast frequency → Positive affect → Life satisfaction	0.133	0.03	4.376	<0.001	[0.073, 0.192]
**Total effects**					
Fruits and Vegetables → Life satisfaction	0.162	0.041	3.948	<0.001	[0.081, 0.242]
Breakfast frequency → Life satisfaction	0.138	0.043	3.237	0.001	[0.054, 0.221]
**Path coefficients**					
Positive affect → Life satisfaction	0.705	0.027	26.245	<0.001	[0.652, 0.757]
Fruits and Vegetables → Life satisfaction	0.047	0.031	1.526	0.127	[−0.013, 0.108]
Breakfast frequency → Life satisfaction	0.005	0.034	0.155	0.877	[−0.06, 0.071]
Fruits and Vegetables → Positive affect	0.162	0.041	3.93	<0.001	[0.081, 0.243]
Breakfast frequency → Positive affect	0.188	0.041	4.613	<0.001	[0.108, 0.268]

***Note.*** β = completely standardized coefficient; SE = Standard error; CI = Confidence interval.

## Data Availability

The original contributions presented in the study are included in the article. Further inquiries can be directed to the corresponding author.

## Abbreviations

The following abbreviations are used in this manuscript:

FV	Fruits and Vegetables
EBS-8	Subjective Well-Being Scale (Escala de Bienestar Subjetivo—8 items)
AVE	Average Variance Extracted
VIF	Variance Inflation Factor
SEM	Structural Equation Modeling
SPSS	Statistical Package for the Social Sciences
WHO	World Health Organization
SD	Standard Deviation
α	Cronbach’s Alpha
CI	Confidence Interval
RMSEA	Root Mean Square Error of Approximation
R^2^	Coefficient of Determination
